# Unusual congenital goiter due to maternal Hashimoto thyroiditis: a case report

**DOI:** 10.3389/fped.2024.1348431

**Published:** 2024-05-22

**Authors:** Laura Català, Judit Casas, Sean Yeh, Maria Josa-Eritja, Mireia Tirado-Capistros, Elisenda Moliner, Gemma Carreras

**Affiliations:** ^1^ Department of Pediatrics, Hospital de la Santa Creu i Sant Pau, Barcelona, Spain; ^2^ Institut de Recerca Biomèdica - IIB Sant Pau, Barcelona, Spain; ^3^ Department of Pediatrics, Obstetrics and Gynecology, and Preventive Medicine and Public Health, Universitat Autònoma de Barcelona, Bellaterra, Spain

**Keywords:** goiter, hypothyroidism, autoantibodies, newborn, congenital hypothyroidism (CH)

## Abstract

Congenital hypothyroidism (CH) is the most common cause of endocrinopathy in the newborn Its incidence lies between 1 in 3,000 and 1 in 2,000, However, congenital goiter is a rare form of presentation. Hypothyroidism secondary to autoimmune etiology is extremely rare, with an incidence of 1:84.700–1:31.000 newborns. Anti-thyroid peroxidase antibodies (TPOAb) are able to cross the placenta but rarely induce hypothyroidism in the newborn, much less goiter. A case of congenital goiter in a male newborn secondary to maternal high TPOAb levels is reported. The mother was diagnosed of Hashimoto thyroiditis prior to the pregnancy. At birth, a grade 3 goiter was detected in the newborn. Laboratory testings revealed hypothyroidism with free thyroxine of 7.6 pmol/L, thyroid-stimulating hormone of 108 mUI/L and high TPOAb levels. Treatment with Levothyroxine was started the second day of life with progressive thyroid function normalization. Neurological development has been normal until the date.

## 1 Introduction

Normal thyroid function during childhood is an essential condition for proper growth and neurodevelopment. Congenital hypothyroidism (CH) is the most common cause of endocrinopathy in the newborn, with an incidence between 1 in 3,000 and 1 in 2,000 (1). It is also considered one of the most common preventable causes of intellectual disability. In addition, most patients with congenital hypothyroidism will not present clinical symptoms at birth or this will be non-specific. Neonatal screening programs were established in the 1970s and are available in 30% of countries worldwide (predominantly developed countries). Congenital hypothyroidism is due to thyroid hormone (TH) defect and can be classified as: primary (thyroidal), central (hypothalamo–pituitary axis) and peripheral. Another classification is: permanent or transient, as well as isolated or syndromic (2). The early treatment of CH is of vital importance for neurological development, starting supplementation with adequate doses of Levothyroxine (T4l) as soon as CH is detected, preferably in the first two weeks of life (1).

Congenital goiter is a rare condition produced by multiple causes such as dyshormonogenesis, transplacental passage of maternal antibodies, maternal ingestion of antithyroid drugs, activating mutations of the TSH receptor, or congenital syndromes (3). Depending on the etiology, goiter can be associated to hypothyroidism, hyperthyroidism or normal thyroid function. Once the goiter is detected, management will depend on the thyroid function and its etiology.

Transient CH due to autoimmune etiology is extremely rare, with an incidence of 1:84.700–1:31.000 newborns (4). Hashimoto's thyroiditis (TH), also known as chronic lymphocytic thyroiditis, is a thyroid autoimmune disease and it’s the most prevalent thyroid pathology in women of childbearing age. Anti-thyroid peroxidase antibodies (TPOAb) are able to cross the placenta but rarely induce hypothyroidism in the newborn, much less goiter (5).

Due to its extremely rare presentation we present a case of congenital goiter secondary to maternal Hashimoto's thyroiditis.

## 2 Case description

We present a case of congenital goiter in a male term newborn secondary to maternal high TPOAb levels.

The mother was a 27-year-old healthy woman and was diagnosed of Hashimoto's thyroiditis 4 years prior to the pregnancy, with positive TPOAb levels (>1,000U/L, external lab test) with normal thyroid function. She presented 3 years prior to the pregnancy subclinical hypothyroidism that did not undergo treatment. Further on, no more thyroid function tests were conducted.

No history of hypothyroidism was reported in the parents’ family. The pregnancy progressed without complications. It was the mother's first pregnancy, with no prior history of miscarriages. She tested negative for syphilis, hepatitis B, hepatitis C, and human immunodeficiency virus (HIV), and was immune to toxoplasma and rubella. Prenatal ultrasounds revealed no morphological abnormalities, except for an initial suspicion of aortic coarctation, which was later ruled out. Additionally, the thyroid gland and fetal vitals were found to be normal. There were no reported maternal infections during pregnancy, and she tested negative for group B streptococcus. The baby was delivered at 40 + 0 weeks gestation with APGAR scores of 7/8/8. Mild distress was noted at birth, which resolved within the first few hours of life.

**Figure 1:**
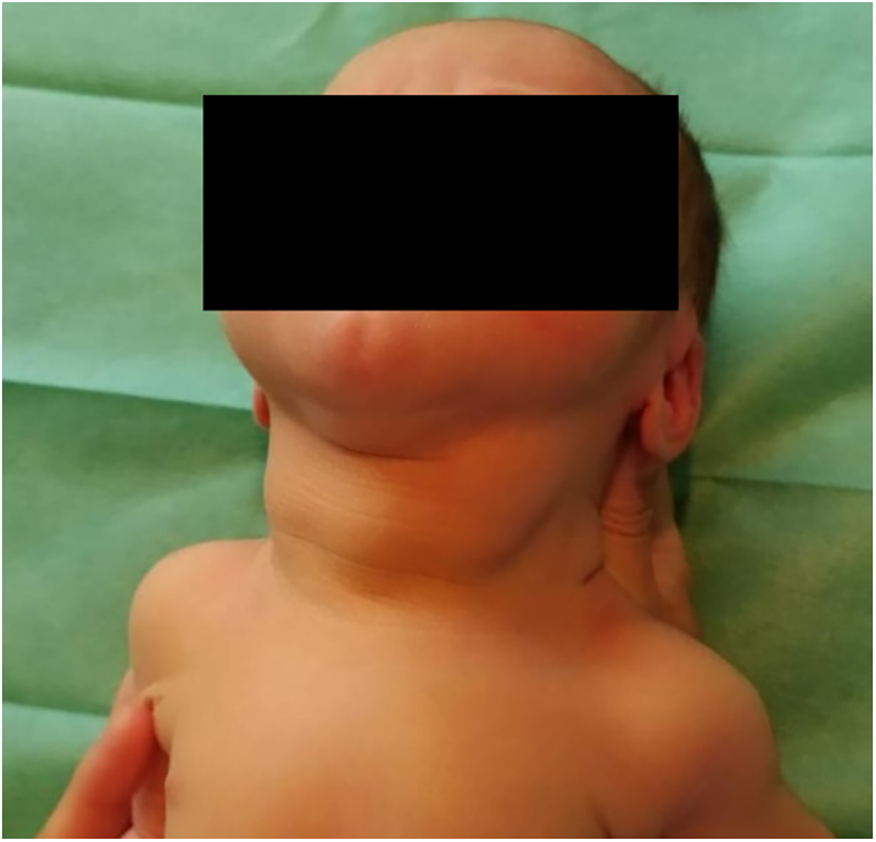
Congenital goiter detected at birth.

At birth, a grade 3 goiter was detected (see Figure 1) in the newborn, non-obstructive and apparently asymptomatic.

## 3 Diagnostic, treatment and follow-up

Given the early detection of the goiter, laboratory testings were conducted in the first day of life, revealing hypothyroidism with free thyroxine of 7.6 pmol/L (reference range for newborn 10–26 pmol/L) and thyroid-stimulating hormone of 108mUI/l (<10 mUI/L in neonates). A thyroid ultrasound (US) was performed the second day of life, showing diffusely enlarged thyroid in both its lobes and the thyroid isthmus. The echostructure appeared homogeneous without nodules or intraparenchymal lesions and the doppler study showed normal vascularization. The estimated size was of 5.76 cm^3^ [normal size in neonates 0.63 ± 0.2 cm^3^ (6)].

A scintigraphy was performed to rule out dyshormonogenesis (7), revealing homogeneous uptake of the radiotracer, which was Technetium-99 m pertechnetate, with no evidence of ectopic thyroid tissue or nodules. The observed pattern was indicative of a diffuse goiter.

Treatment with Levothyroxine 15 mcg/kg/day was started the second day of life.

In the etiological study iodine deficiency was ruled out and negative genetic test result was obtained (clinical exome sequencing with human phenotype ontology directed analysis related to goiter, dyshormogenesis and hypothyroidism, including 105 genes). Thyroid autoimmunity was analyzed on the newborn, with high level of TPOAb: 1,842,5 UI/ml (normal <100 UI/ml) with negative anti-thyroglobulin (TgAb) and anti-thyroid hormone receptor antibodies (TSHrAb). Also the mother was studied, remaining euthyroid but presenting at that moment high TPOAb levels (6,500 UI/ml), confirming the diagnosis of hypothyroidism by maternal Hashimoto disease. The mother was referred to the endocrinology department and has been followed-up regularly.

The patient's clinical course showed favorable progression, characterized by gradual normalization of thyroid hormone levels and complete resolution of the goiter within the first month of life. Due to communication issues, the hospital did not receive the positive newborn screening results for congenital hypothyroidism (CH) until the patient was three weeks old and had already begun treatment.

Our patient underwent regular thyroid function assessments every 3–4 weeks. During this period, the dosage of levothyroxine was gradually reduced by 50% until the substitution therapy was discontinued at three months of age (Table 1). At the time of discontinuation, the patient's thyroid function values were as follows: TSH 0.82 pmol/L and T4 16 mUI/L. Current guidelines recommend initial laboratory follow-up evaluations 1–2 weeks after initiating LT4 treatment, with subsequent evaluations every 2 weeks until normalization of TSH levels. Once TSH levels are within the target range, the optimal frequency of follow-up can be extended to every 1–3 months.

**Table 1:** Treatment adjustment according to thyroid hormone levels.

Newborn age	12 h of life	2 days of life	5 days of life	20 days of life	48 days of life	2 months and 10 days	3 months	4 months	8 months	1 year
TSH levels (mU/L)	108	23.3	3.08	0.24	0.16	0.27	0.82	0.84	1.06	3
Reference values (−2 SD to +2 SD) (18):	2.43–24.03	1.4–13.1	0.6–6.82	0.58–5.57	0.58–5.57	0.58–5.57	0.58–5.57	0.58–5.57	0.58–5.57	0.57–5.54
Free-LT4 Levels (pmol/L)	7.6	9.5	17.4	20.4	18.6	16.9	16.0	14.7	15.0	18.4
Reference values (−2 SD to +2 SD) (18):	12.12–56.54	12.12–56.54	12.32–42.54	12.81–44.33	12.81–44.33	13.41–36.83	13.41–36.83	13.82–31.39	13.82–31.39	14.14–28.22
Levothyroxine dosage (µg/kg/day)		15	15	10.5	6	3	End of treatment	No treatment needed	No treatment needed	No treatment needed

SD, standard deviation.

A follow-up ultrasound was conducted at three months of age: it showed significant volume reduction. Monitoring of thyroid peroxidase antibodies (TPOAb) was not conducted during treatment, as the primary objective was to achieve euthyroidism and authors have not reported a relation between TPOAb levels and thyroid function (8).

Notably, the patient did not exhibit any clinical signs of hypothyroidism prior to treatment initiation, and neurological development has remained normal to date (currently at three years of age).

## 4 Discussion

### 4.1 Congenital hythyroidism

CH poses a significant health concern due to its potential for adverse neurological outcomes if left undetected and untreated. The condition often presents with nonspecific or absent clinical manifestations at birth, attributed in part to the trans-placental transfer of maternal thyroid hormones, leading to missed diagnoses in up to 95% of cases (9).

The etiology of congenital hypothyroidism (CH) often dictates its clinical course, determining whether hypothyroidism is transient or permanent and whether it is primary, secondary, or peripheral. In iodine-sufficient regions, thyroid dysgenesis accounts for approximately 85% of CH cases, while dyshormonogenesis typically explains 10%–15% of cases. Less common causes include maternal autoimmune thyroid disease.

### 4.2 Newborn screening (NBS) and diagnose

NBS for CH is imperative for timely detection and intervention. The primary goal of NBS is to identify all forms of primary CH, ranging from mild to severe presentations (1). Thyrotropin (TSH) measurement emerges as the most sensitive screening tool for primary CH. However, it's crucial to recognize that NBS alone may not completely eliminate the risk of false negatives, and it does not detect central CH, emphasizing the need for clinical vigilance and consideration of hypothyroidism in the presence of relevant symptoms (10).

Since 1982, congenital hypothyroidism (CH) has been tested in all newborns born in Spain, with the primary strategy focusing on newborns’ TSH levels to detect primary hypothyroidism. If TSH levels exceed the 99th percentile, the result is considered positive, with newborns having TSH levels >20 uUI/ml requiring immediate diagnosis and treatment. Conversely, positive screenings with TSH levels <20 uUI/ml will need to be retested before 15 days of life (11).

It is important to emphasize the importance of a thorough physical examination in order to detect alterations in the thyroid gland early. The examination of the thyroid in the newborn will be carried out with the baby in the supine position and cervical hyperextension, although the goiter size can be overestimated in some cases.

In accordance with current guidelines regarding congenital hypothyroidism, it is advised that either ultrasonography (US), scintigraphy, or a combination of both modalities be employed for diagnostic evaluation in neonates (1). In our clinical scenario, initial assessment utilized ultrasonography due to its accessibility. However, it is widely acknowledged that scintigraphy is the most accurate diagnostic test for determining the etiology of CH, especially in case of thyroid dysgenesis. Our approach integrated both thyroid ultrasonography and scintigraphy, facilitating the acquisition of high-resolution anatomical and functional insights.

### 4.3 Maternal autoimmune thyroid disease

Maternal autoimmune thyroid disease presents unique considerations in the context of CH. The prevalence of autoimmune hypothyroidism or Hashimoto disease, and autoimmune hyperthyroidism or Graves disease in women are approximately 0.3% and 0.5%, respectively but CH secondary to autoimmune etiology is extremely rare, with an incidence of 1:84.700–1:31.000 newborns (12). Evidence suggests that pregnant women with autoimmune thyroid disease should undergo strict thyroid function control and therapy has to be initiated if hypothyroidism is detected (13).

Our case is exceptional since several authors agree that despite TPOAb being able to cross the placenta, it does not usually influence fetal or neonatal thyroid function (14, 15). According to Marín et al. study, most offspring of mothers with Hashimoto's thyroiditis had normal thyroid function with the most frequent alteration being high TSH with normal T4 levels. They only report one premature baby with persistent hyperthyrotropinemia that required replacement therapy with levothyroxine (16).

In cases of maternal hypothyroidism or hyperthyroidism, systematic thyroid function screening in newborns is recommended, including LT4 and TSH. However, if maternal thyroid function remains normal during pregnancy, routine neonatal thyroid blood testing may not be warranted, and regular NBS may be sufficient, similar to standard procedures for other newborns. Additionally, whereas congenital goiter can be observed in autoimmune hyperthyroidism, it is exceptionally rare in autoimmune hypothyroidism. In our particular case, despite the mother remaining euthyroid, neonatal screening wouldn't have been indicated. However, once goiter was detected, considering the maternal medical history, autoimmune etiology should have been prioritized, highlighting the importance of clinical judgment in such cases and the need for further research to guide modifications in current screening guidelines.

### 4.4 Treatment and prognosis

Early initiation of levothyroxine treatment is paramount for optimizing neurological outcomes in infants with CH (17). Adverse effects of long-term levothyroxine treatment are rare when appropriately prescribed. The recommended initial dosage is 10–15 mcg/kg/day, with adjustments based on the severity of hypothyroidism (1).

The goal of L-T4 treatment is to support normal neurocognitive development and growth. Dose adjustments of hormone supplementation are recommended until the recovery of the thyroid axis is verified, which in some cases can be delayed for up to 3 years. Subsequently, a close follow-up with analytical determinations will be necessary in the event of the slightest suspicion of recurrence of hypothyroidism (8). With proper management, hypothyroidism secondary to maternal autoimmune disease typically resolves within 3–5 months as maternal antibodies dissipate, leading to a positive long-term prognosis.

In conclusion, the management of congenital hypothyroidism secondary to maternal autoimmune disease necessitates an early detection and proper endocrinologist management. CH secondary to TPOAb still needs to be properly evaluated and characterized. With vigilant clinical care and adherence to established guidelines, the prognosis for infants with CH is promising, emphasizing the importance of ongoing monitoring and support.

## Data Availability

The original contributions presented in the study are included in the article/Supplementary Material, further inquiries can be directed to the corresponding author.
